# Do the implementation processes of a school-based daily physical activity (DPA) program vary according to the socioeconomic context of the schools? a realist evaluation of the *Active at school* program

**DOI:** 10.1186/s12889-022-12797-7

**Published:** 2022-03-03

**Authors:** Véronique Gosselin, Suzanne Laberge

**Affiliations:** School of Kinesiology and Physical Activity Sciences, Université de Montréal, C.P. 6128, Succursale Centre-Ville Montréal, H3C 3J7 Québec, Canada

**Keywords:** School-based physical activity, Realist evaluation, Context, Socioeconomic status, Community empowerment, School health, Health policy

## Abstract

**Background:**

Less than half of Canadian children meet the Canadian Physical Activity (PA) Guidelines, and the proportion is even lower among children living in underprivileged neighbourhoods. Regular PA supports physical, cognitive, and psychological/social health among school-aged children. Successful implementation of school-based daily physical activity (DPA) programs is therefore important for all children and crucial for children who attend schools in lower socioeconomic settings. The purpose of this study is to uncover what worked, for whom, how, and why during the three-year implementation period of a new “flexible” DPA program, while paying particular attention to the socioeconomic setting of the participating schools.

**Methods:**

This study is a realist evaluation using mixed methods for data generation. Longitudinal data were collected in 415 schools once a year during the three-year implementation period of the program using questionnaires. Data analysis was completed in three steps and included qualitative thematic analysis using a mixed inductive and deductive method and chi-square tests to test and refine context-mechanism-outcome (CMO) configurations.

**Results:**

Giving the school teams autonomy in the choice of strategies appropriate to their context have allowed schools to take ownership of program implementation by activating a community empowerment process, which resulted in a cultural shift towards a sustainable DPA provision in most settings. In rural underprivileged settings, the mobilization of local resources seems to have successfully created the conditions necessary for implementing and maintaining changes in practice. In disadvantaged urban settings, implementing local leadership structures (leader, committee, and meetings) provided pivotal assistance to members of the school teams in providing new DPA opportunities. However, without continued external funding, those schools seem unable to support local leadership structures on their own, jeopardizing the sustainability of the program for children living in disadvantaged urban areas.

**Conclusion:**

By exploring CMO configurations, we have been able to better understand what worked, for whom, how and why during the three-year implementation period of the *Active at School!* program. When implementing DPA policies, decision makers should consider adjusting resource allocations to meet the actual needs of schools from different backgrounds to promote equal PA opportunities for all children.

**Supplementary Information:**

The online version contains supplementary material available at 10.1186/s12889-022-12797-7.

## Background

Less than half of Canadian children meet the physical activity (PA) recommendation within the *Canadian 24-Hour Movement Guidelines for Children and Youth* [1], and the proportion is even lower for children living in underprivileged neighbourhoods [2]. Yet, regular PA supports physical, cognitive, and psychological/social health among school-aged children [3]. Schools are an ideal setting in which to increase PA among young people, as almost all children from all socioeconomic status (SES) backgrounds can be reached during critical periods of development [4]. Moreover, results of meta-analyses showed positive effects of active classrooms on academic achievement [5, 6] and positive effects on classroom behaviour [7]. Since 2005, several Canadian provinces [8] have adopted school-based daily PA (DPA) policies. These policies can be referred to as universal policies [9] because they aim to create supportive environments for PA that are universal to all children. In 2017, the province of Quebec joined these jurisdictions by launching its first policy on sports, PA and leisure, which included legislative provisions mandating the integration of 60 min of DPA within all elementary schools (K-Grade 6) by 2022 [10]. To support the schools in their DPA implementation, a specific program, *Active at school!* (*À l’école, on bouge!* [Measure 15023]) was concomitantly launched [11]. This program provides participating schools financial resources over three years to implement opportunities for students to be active 60 min every school day. It adopts a “flexible” approach to DPA, meaning that the participating schools are provided with the autonomy to develop their own custom action plan and select the new practices that are appropriate for their context and needs [11].

Given the overall rates of physical inactivity and given that socioeconomic disparities affect the activity level of children [1, 2], successful implementation of universal DPA policies is important for all children and crucial within lower-SES schools. However, a recognized limit of universal policies, including those implemented in schools, is that they are generally not effective in reducing inequalities [12–16]. It is possible that school-based DPA policies provide an advantage to children attending schools that are already in a favourable position or fail to proportionately improve the outcomes of those in less favourable settings. This situation would result in widening health inequalities [17].

Several factors within and outside the school environment have been found to hinder adoption, implementation, and sustainability of school-based PA programs [18–20] but, to our knowledge, no study has documented whether and how the implementation of school-based DPA programs might vary based on schools’ SES context. The purpose of this study is therefore to evaluate the implementation of the DPA program *Active at School!* while paying particular attention to the socioeconomic setting of the schools to better understand whether that setting affects implementation and, if so, how.

Realist evaluation [21] offers an interesting approach for achieving this objective. A realist evaluation aims at providing theory-driven explanations of how complex programs work within the context of their implementation [21, 22]. As a form of theory-driven evaluation, the realist evaluation develops tentative initial program theories about how a program works. These initial theories generally combine elements of substantive theories with stakeholders’ assumptions about how and why the program may work, derived from their research and/or professional expertise. These realist theories are used to reveal the underlying logic of programs and are then tested and refined empirically through data collection and analysis [23]. A central tenet of the realist approach is that programs work differently in different contexts. In a realist evaluation, the outcome of a program can be explained by the action of specific mechanisms in specific contexts. Realist inquiry is concerned with identifying the underlying mechanisms through which outcomes occur (or do not), and the contexts in which those mechanisms are triggered. Pawson and Tilley [21] name these configurations “context-mechanism-outcome configurations” (CMO configurations). Given that the school teams participating in the *Active in School!* program had the autonomy to develop action plans tailored to their needs and adapted to their context, conducting a realist evaluation is highly relevant to better understand what worked (or did not), through which mechanisms and in which socioeconomic context.

The purpose of this study is therefore to evaluate the implementation of the DPA program *Active at School!* using the realist evaluation approach. More precisely, we intend to better understand what worked (and did not work), for whom, how, and why during the three-year implementation period of the DPA program in participating schools, while paying particular attention to the socioeconomic context of the schools. Hence, this study provides a better understanding of the underlying mechanisms at play during implementation of school-based DPA and inform the development of future health-related policies in schools that might contribute to the reduction of social inequalities.

## Methods

### Participants and program description

The participants in this study were all the schools (415) composing the first cohort of the *Active at school!* program initiated in 2017 [11]. *Active at school!* aims to financially support schools, over a three-year period, to help them implement opportunities for the students to be active 60 min every school day, including during physical education (PE) classes. Financial resources are allocated on a degressive basis over the three-year period (during their first year of participation, schools received, in average, 15 614$ (SD = 8 114$) and this amount dropped to an average of 6 400$ (SD = 3 567$) during their third year of participation). Schools participated on a voluntary basis, and regional school boards oversaw the selection of the schools and the allocation of the financial resources provided by the Ministry. The resources allocated were to be used by selected school teams to implement new practices, both at the level of the school organization (e.g., set up a committee, appoint an in-school leader, or other) and the interventions themselves (e.g., schedule in-class active breaks or lead physical activities during recess). The participating schools were free to develop their own action plans and select the new practices that were appropriate for them. Schools nonetheless had access to supportive counselling from the academic advisor of their regional school board, as well as access to tools and ideas (e.g., brain-break videos and suggestions for classroom PA and active corridors) provided by *Force 4* [24], a tool kit for schools developed by a public Foundation dedicated to the promotion of PA in Quebec. In the first year of implementation in 2017, a maximum of 450 participating schools were fixed by the Ministry, and 415 joined the program. These 415 schools reached the end of the three-year funding period in 2020.

### Study design

This study is a realist evaluation [21] using mixed methods for data generation [25]. Longitudinal data were collected once a year throughout the three-year funding period of the program to document the implementation processes in the participating schools and to gather information on stakeholder perceptions of how and why the program “worked.” The use of a mixed-methods approach was deemed relevant for two main reasons. First, qualitative data were collected to explore and refine CMO configurations as they provided stakeholders’ perceptions about what worked, in their context, and why. Second, the use of quantitative data allowed a large amount of information to be collected from all participating schools throughout the three-year intervention period, which would not have been possible using only qualitative data for logistic reasons (i.e., the lack of human and financial resources to collect and analyze qualitative data from 415 schools). The mixed-methods approach can have different designs depending on how qualitative and quantitative approaches are combined [25], and we used a triangulation design to obtain different but complementary data to arrive at the best understanding of the research problem.

Ethical approval for the study was obtained from the (author’s institutional affiliation) Multifaculty Ethics Board. We followed RAMESES II standards for realist evaluations and the stages of realist evaluation including theory formulation, theory testing and refining by exploring the complex interactions of contexts, mechanisms and outcomes (CMO configurations) [26].

### Theory formulation

“Realist theories typically combine elements of substantive theories with stakeholders’ theory – i.e. their ideas about how programmes may work” ([27], p. 2). These realist theories are used to reveal the underlying logic of programs and are then tested and refined using the CMO configurations [26].

The Quebec Ministry of Education’s (MEQ) ideas about how *Active at school!* may work is that by allocating resources and allowing the schools to develop a custom plan and implement actions tailored to their needs, the program would favour a shift in the school culture towards a sustained provision of DPA [28]. This logic is akin to bottom-up, community-based approaches to policy-making [29, 30] as the implementation of the program is based on the mobilization of a group (a school team), whose members come together to propose actions tailored to their own context to initiate a change of practices. In theory, the school team is therefore more likely to “take ownership” of the program, facilitating DPA sustainability and longer-term beneficial health and academic outcomes for the students [28].

The process by which a community can bring about cultural and structural changes eventually leading to improvement in health outcomes has been designated in the health promotion literature as community empowerment [30, 31]. Laverack and Labonté [30] argue that community empowerment can be monitored by tracking nine “domains”: how a program 1) improves participation; 2) develops local leadership; 3) builds organizational structures; 4) increases problem assessment capacities; 5) enhances critical awareness; 6) improves resource mobilization; 7) strengthens links to other organizations and people; 8) creates an equitable relationship with outside agents; and 9) increases control over program management. This robust and reliable approach has been applied in different program and cultural contexts to better understand how a group progresses towards more organized forms of social action [30].

To uncover the underlying logic of the *Active at school!* program, we combined the MEQ’s theory about how the program works and the community empowerment conceptual framework, using its nine domains. Each school team represents a small community comprising the principal, teachers, physical education (PE) teachers, and daycare staff. The nine domains of community empowerment are the potential mechanisms, potentially triggered differently in different contexts, by which the program would lead to the potential outcome—the intended shift in the school culture towards sustained DPA provision. Table 1 summarizes the various potential CMO configurations. Since, as far as we know, the concept of community empowerment has never been applied in a school-based PA setting, we used a recent systematic review [18] that identified factors associated with implementation of school-based PA interventions in a real-world setting to clarify how each domain could manifest itself concretely in a school setting. Furthermore, to operationalize the potential shift in the school culture, we combined the MEQ’s assumptions [28] and Schein’s definition of culture [32]. Finally, we considered two main contexts: the socioeconomic setting of the schools (high, middle or low) and the geographic setting (rural or urban). Geographic setting was selected as a primary context, together with socioeconomic setting, because of substantial differences in PA between rural and urban settings [33]. To refine the study of these main contexts, contextual factors reported in Cassar et al. [18] as influencing the implementation of school-based PA programs were also considered (Table 1). Taken together, the contextual factors, mechanisms and outcomes presented in Table 1 offer CMO configuration assumptions that potentially explain what worked, for whom, how and why during the implementation of the *Active at school!* program. For instance, it is possible that contextual factors such as school size, physical factors or staff turnover differ based on socioeconomic and/or geographic setting, making it easier (or harder) for a school team to use the resources allocated by the program to build organizational structures such as a committee (mechanism), which, in turn, might lead to divergent DPA routine implementation or staff engagement (outcomes). The initial buy-in to PA within a school (contextual factor) could also influence how the program improves participation (mechanism) and, eventually, the extent to which the school implements new PA practices (outcome).

**Table 1:** Framework of potential CMO configurations underlying the *Active at school* program

CONTEXT	MECHANISM			OUTCOME	
Potential contexts and contextual school-level factors [18] affecting implementation	Potential mechanism (domains of community empowerment [30])	Definition [30]	Potential manifestation of the mechanism in a school setting [18]	Potential outcome	Potential manifestation of outcome (MEQ [27] and authors’ assumptions based on Schein’s definition of culture [32])
*Context* Socioeconomic setting Geographic setting *Contextual factors* General organizational factors Organizational norms Specific staffing considerations Classroom management and Disruptive student behaviours Perceived need for innovation Funding Characteristics of the school (school size; language barrier; student ethnicity; built environment) Staff turnover/changing roles Physical factors (e.g., appropriate footwear/clothing for students)	Improves participation	The extent to which community members are involved in activities and decisions on planning and implementation	Student engagement and motivation Shared decision-making	Shift in the school culture towards a sustained DPA provision	Changes in practice (implementation of new DPA routines in class, at the school, during recess and at the daycare services) Change in the school team’s perceived value of PA for its contribution to academic success Changes in the commitment level of school team members to daily active time
	Develops local leadership	The extent to which leaders are taking initiative, with support from their organizations and work with outside groups to gain resources	Communication Formulation of tasks Leadership Program champion		
	Increases problem assessment capacities	The extent to which the community identifies problems, solutions and actions and uses assessment to strengthen community planning	Observed benefits of innovation Perceived benefits of innovation Perceived need for innovation Shared vision		
	Enhances critical awareness	The extent to which community groups have the ability to self-analyze and improve their efforts overtime, leading to collective change			
	Improves resource mobilization	The extent to which resources are raised and community decides on distribution			
	Strengthens links to other organizations and people	The extent to which links are defined and organizations involved in community development, based on mutual respect and generating resources and finances, leading to improvements for the community	Coordination with other agencies Parent support and perceptions		
	Builds organizational structures	The extent to which organizations have established links with each other within the community and have mechanisms to allow their members to provide meaningful participation			
	Creates an equitable relationship with outside agents	The extent to which the community makes decisions with the support of agents. Agents facilitate change through training and support and act on behalf of the community to build capacity			
	Increases control over program management	The extent to which the community self-manages planning, policy and evaluation with limited assistance from agents, developing sense of community ownership			

### Data collection

Online questionnaires were sent to all participating schools through the MEQ’s accountability platform once a year throughout the program’s funding period (at the end of the school year, in May 2018, 2019 and 2020). Principals and, where appropriate, in-school appointed program leaders, were asked to jointly complete the questionnaires. In accordance with the realist evaluation methodology [21], the questionnaires were built to document the implementation processes of the program in the participating schools and to gather information on stakeholder perceptions about how and why the program “works.” The questionnaires contained an average of 40 closed-ended questions and 15 open-ended questions covering all of the configurations presented in Table 1. More specifically, the YEAR 1 questionnaire was designed to document the program’s initial implementation in terms of contextual factors, mechanisms and changes in practices. The YEAR 2 questionnaire documented modifications to the changed practices, as well as the impact of staff turnover, while the YEAR 3 version ascertained the mechanisms involved in maintaining the changed practices, in other words, the shift in school culture as funding came to an end. Two elementary school academic advisors and the program manager at the MEQ assessed the face validity of all questions and answer choices. The questionnaires were adjusted accordingly.

The study results are based mainly on data collected through the YEAR 3 questionnaire. Data from the first two questionnaires were nonetheless used to better define contextual aspects in order to enhance the analyses of data collected in YEAR 3.

### Data analysis

The data collection yielded a large quantity of qualitative data (answers to the open-ended questions) used in the qualitative analyses. The answers to the closed-ended questions served to create variables used in the quantitative analyses: 6 variables pertaining to potential contextual factors, 12 variables associated with potential mechanisms, and 11 variables related to potential outcomes (Table 2).

**Table 2:** Context- mechanism- and outcome- related variables for quantitative analysis

	Variables	Measures	Categories
Variables related to context	Socioeconomic setting of the school	School-level SES calculated by Quebec Ministry of Education [34]	“High SES”; “Middle SES”; “Low SES”
	Geographic setting of the school	School postal code	“Rural”; “Urban”
	Number of students at the school	Questionnaire, year 1, closed-ended question	NA – continuous variable
	Teacher and PE teacher turnover	Questionnaire, year 2, closed-ended question	“Yes, major turnover (≥ 25%)”; “Yes, minor turnover (< 25%)”; “No”
	School-team resistance encountered	Questionnaire, year 1, closed-ended question	“Yes, from more than half the members”; “Yes, from about half the members”; “Yes, from a minority of members”; “No”
	Per-student financial amount received	Questionnaire, year 1, closed-ended question	NA—continuous variable
Variables related to mechanisms	Students involved in implementation	Questionnaire, year 3, closed-ended question	“Yes”; “No”
	Student participation	Questionnaire, year 3, closed-ended question	“Yes”; “No”
	Educational plan modified	Questionnaire, year 3, closed-ended question	“Yes”; “No”
	Champions recognized	Questionnaire, year 3, closed-ended and open-ended questions	“Yes”; “No” and qualitative data
	Intention to maintain the leader’s role	Questionnaire, year 3, closed-ended question	“Yes”; “No”; “NA (we didn’t have a designated leader)”
	Intention to maintain a DPA committee	Questionnaire, year 3, closed-ended question	“Yes”; “No”; “NA (we didn’t form a committee)”
	Intention to maintain formal meetings about DPA	Questionnaire, year 3, closed-ended question	“Yes”; “No”; “NA (we didn’t hold formal meetings)”
	Positive/negative changes in students observed	Questionnaire, year 3, closed-ended question	“Yes”; “No”
	Strategies for finding alternative sources of funding	Questionnaire, year 3, closed-ended question	“Yes”; “No”
	Strategies for supporting new staff	Questionnaire, year 3, closed-ended and open-ended questions	“Yes”; “No” and qualitative data
	Strategies for updating activities	Questionnaire, year 3, closed-ended and open-ended questions	“Yes”; “No” and qualitative data
	Partnerships maintained	Questionnaire, year 3, closed-ended question	“Yes”; “No”; “NA (we didn’t form any new partnerships)”
Variables related to outcomes	*New in-class PA routines integrated*		
	Active breaks	Questionnaire, year 3, closed-ended question	“Yes”; “No”
	Active learning activities	Questionnaire, year 3, closed-ended question	“Yes”; “No”
	*New PA routines integrated at the school*		
	Active recesses	Questionnaire, year 3, closed-ended question	“Yes”; “No”
	Active corridors	Questionnaire, year 3, closed-ended question	“Yes”; “No”
	Active assemblies	Questionnaire, year 3, closed-ended question	“Yes”; “No”
	Outdoor field trips	Questionnaire, year 3, closed-ended question	“Yes”; “No”
	New PA routines integrated at daycare services	Questionnaire, year 3, closed-ended question	“Yes”; “No”
	Change in the perceived value of PA for its contribution to academic success	Questionnaire, year 3, closed-ended and open-ended questions	“Yes”; “No” and qualitative data
	Change in the commitment of homeroom teachers to daily PA	Questionnaire, year 3, closed-ended and open-ended questions	“Increased”; “No change”; “Decreased” and qualitative data
	Change in the commitment of PE teachers to daily PA	Questionnaire, year 3, closed-ended and open-ended questions	“Increased”; “No change”; “Decreased” and qualitative data
	Change in the commitment of daycare staff to daily PA	Questionnaire, year 3, closed-ended and open-ended questions	“Increased”; “No change”; “Decreased” and qualitative data

The data analysis was organized into three major phases and focused on realist theory testing and refinement. Phase 1 was designed to provide more specific answers to the question of *what worked* and *how?* while phases 2 and 3 looked at *what worked, for whom* and *why?*

1. A qualitative thematic analysis was undertaken to code the qualitative data in a mixed inductive and deductive manner [35]. The starting point for the thematic analysis was the framework concepts (Table 1), with a focus on mechanisms. First, the lead author read all data to become familiar with participant responses. Second, the qualitative data were divided according to their geographical and socioeconomic settings and analyzed separately. Sub-categories were generated inductively and deductively, using the framework concepts (Table 1), to assign meaning to portions of text within each setting. Subcategories relating to similar concepts were grouped into larger categories and compared between settings to identify similarities and differences. Subcategories and categories were reviewed by the corresponding author. This first step allowed the identification of the broad mechanisms at play (MO links) and a first exploration of CMO links.
2. Next, quantitative analyses were undertaken to better understand *what worked, for whom,* and *why*? Chi-square tests were used to examine differences in outcome variables based on the two main contextual variables (geographic and socioeconomic setting) to better understand *what worked for whom*. Interactions between geographic and socioeconomic context were examined and results are presented according to socioeconomic context for rural and urban schools separately when there was an interaction between the two contexts. Next, the same analyses were used with mechanism variables and contextual factor variables to better understand *why*. We used these quantitative results to refine CMO configurations identified in the previous step and explore new CMO configurations.
3. A last qualitative thematic analysis was undertaken to refine the CMO configurations identified in steps 1 and 2. This step involved searching for context, mechanism and outcome elements and patterns across the qualitative data collected in a mixed inductive and deductive manner.

**Fig. 1:**
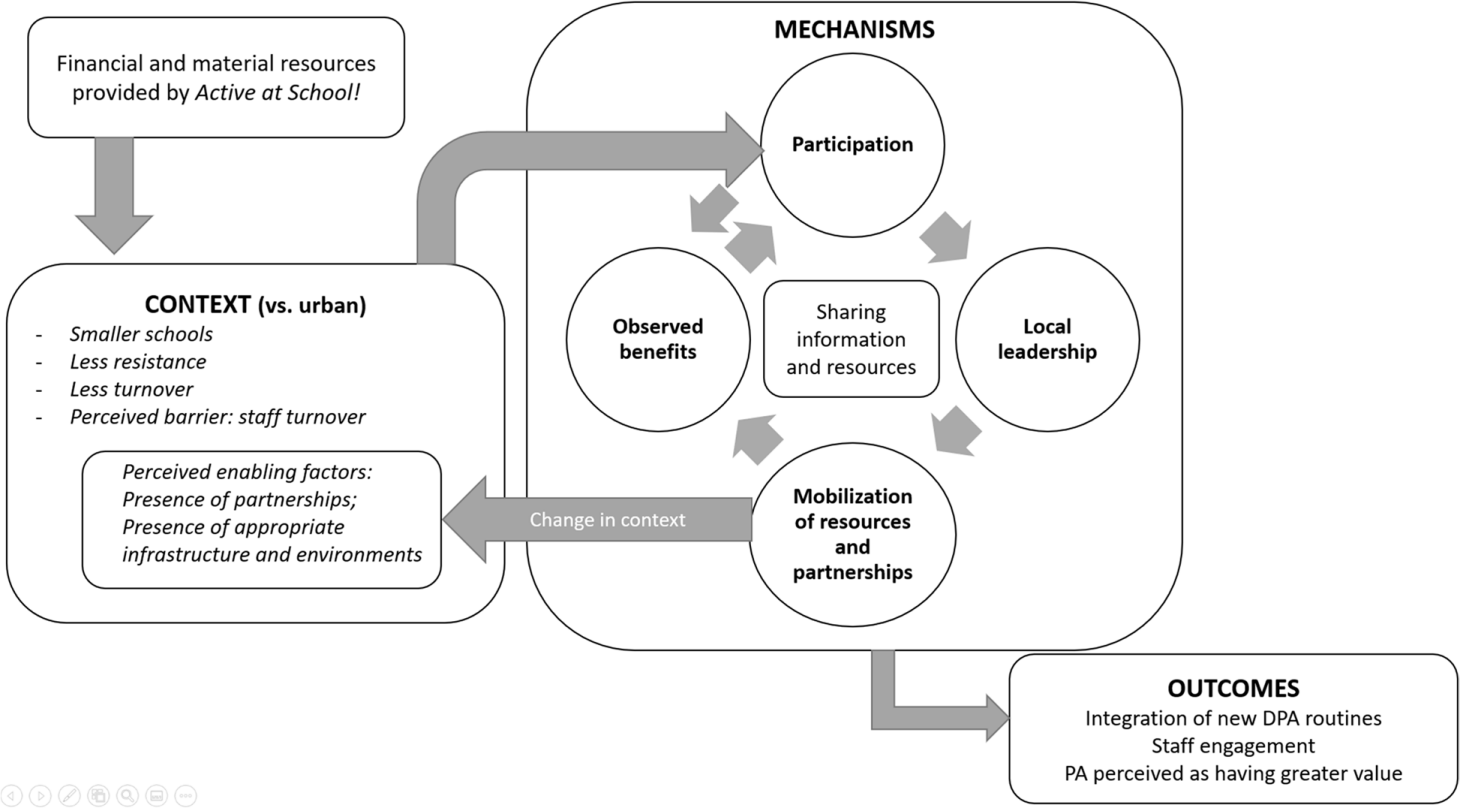
Interaction between CMOs in low SES rural setting

**Fig. 2:**
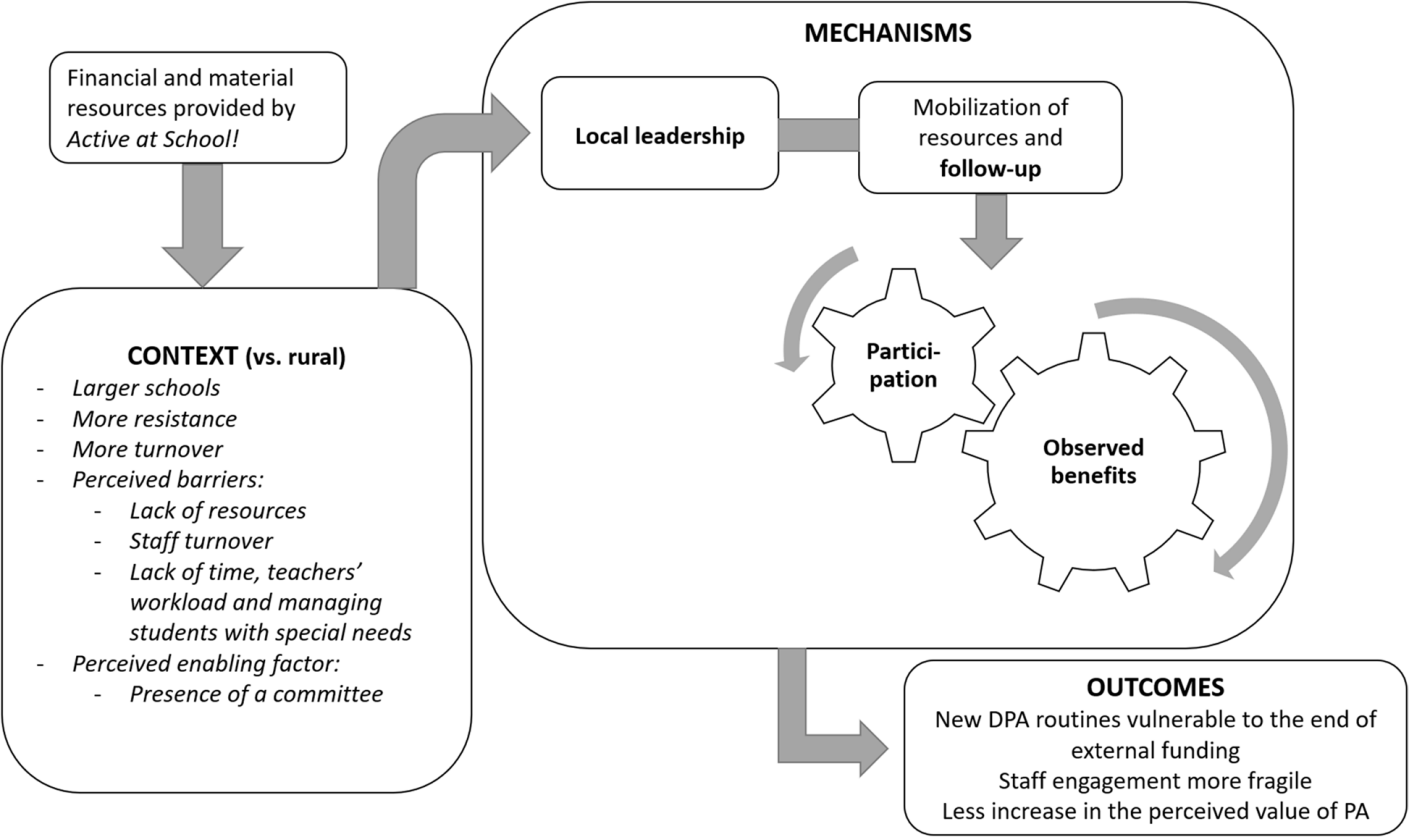
Interaction between CMOs in low SES urban setting

Finally, we summarized the main results in two figures to provide visual representations of the main CMO configurations identified in Phases 1 to 3. These figures (Figs. 1 and 2) are presented in the Discussion alongside a summary of our findings and a comparison with existing literature.

## Results

### Participants

A total of 389 participating schools (out of 415) completed the YEAR 3 questionnaire and were included in the analyses. Of those schools, 36% are located in an underprivileged area, while 68% are in an urban setting (Table 3).

**Table 3:** Participating schools’ characteristics and contextual factors based on socioeconomic and geographic settings

	% of all schools	% of schools by socioeconomic setting			% of schools by geographic setting	
**Contextual factors**		**High SES**	**Middle SES**	**Low SES**	**Rural**	**Urban**
	(*N* = 389)	(*N* = 89)	(*N* = 159)	(*N* = 141)	(*N* = 125)	(*N* = 264)
**Socioeconomic**						
* High SES*	22.7	-	-	-	11.7	27.8**
* Middle SES*	41.4	-	-	-	47.5	38.6
* Low SES*	35.9	-	-	-	40.8	33.6
**Geographic**						
*Rural*	32.5	16.3^a^	36.3^b^	36.0^b**^	-	-
*Urban*	67.5	83.7^a^	63.7^b^	64.0^b**^	-	-
**School size**						
* Number of students,* * mean (SD)*	317 (181)	398 (189) ^a^	310 (169) ^b^	276 (175) ^b***^	187 (117)	375 (177)***
**School-team resistance to the program**						
* No resistance*	53.2	49.5	53.4	55.8	65.6	47.5***
* Yes, from a minority* * of members*	41.6	44.1	41.6	40.1	27.3	48.2***
* Yes, from about* * half the team*	3.2	3.2	3.7	2.0	4.7	2.5
* Yes, from a majority* * of members*	2.0	3.2	1.2	2.0	2.3	1.8
**Resources allocated**						
* Amount ($) per student* * in YEAR 1, mean (SD)*	59.07 (31.25)	52.46 (31.75)	60.46 (32.63)	59.79 (25.98)	72.61 (38.96)	52.78 (24.59)***
**Staff turnover**						
* Large turnover (≥ 25%)* * of homeroom teachers*	19.3	19.2	15.0	23.4	14.2	21.8
* Large turnover (≥ 25%)* * of PE teachers*	33.5	34.6	36.4	29.7	35.4	32.6

*SES*  socioeconomic status,* N*  number of participants, *SD*  standard deviation. * indicates a significant difference (*p*-value < 0.05) between groups; ** indicates a significant difference (*p*-value < 0.01) between groups; *** indicates a significant difference (*p*-value < 0.001) between groups. In the case of differences according to socioeconomic settings, values on the same line with different superscripts (a, b) differ significantly at* p* < 0.05.

### What worked, and how?

The content analysis revealed 12 mechanism subcategories triggered by the program, leading to 3 outcomes categories. The 12 mechanism subcategories were grouped into 4 categories pertaining to 7 of the 9 community empowerment domains. These 4 mechanism categories are presented in Table 4, together with their respective subcategories, ordered from the most to the least frequently mentioned, with the proportion of participating schools reporting the mechanism (for at least one subcategory). The three outcome categories are related to the potential outcomes put forward in the framework (Table 1): implementation of new PA routines (in class, during recess, at the school and/or at the daycare services); commitment level of school team members to active time; and greater perceived value of PA for its contribution to academic success.

**Table 4:** Mechanisms of change and MO links

Mechanism categories (referring to community empowerment domains) and subcategories	% of schools (*N* = 389 *N*_rural_ = 125; *N*_urban_ = 264 *N*_high SES_ = 89; *N*_middle SES_ = 159; *N*_low SES_ = 141)	Salient Extracts of MO links
**Problem assessment capacities** and **critical awareness** *2 subcategories:* -*Experiencing positive changes in students raises awareness of the importance of DPA* *-Sharing positive changes raises critical awareness among staff members*	Total = 44% R = 49% U = 42% H = 47% M = 46% L = 40%	*“The increased level of engagement on the part of the homeroom teachers comes from their students’ engagement in active classes and the benefits provided (reduced need to manage a class of students with a lot of energy, pleasure in learning, collaboration among peers*, etc*.).”* RH-6^a^ *“The teaching staff recognize how beneficial in-class active breaks are for students. The positive aspects are obvious to homeroom teachers, and so they tend to plan activities themselves and don’t mind the lost teaching time.”* UH-162 *“Staff members see the positive effect of active time on students’ concentration*.” RM-38
**Local leadership** (including the leadership of new **organizational structure**) *3 subcategories:* -*Leadership by the principal* -*Leadership by the designated leader* -*Leadership by a committee*	Total = 35% R = 28% U = 39% H = 42% M = 32% L = 35%	*“The staff’s engagement was supported by the leadership of the principal, who insisted that we be more active and that we invest the funds needed to meet the targets in the educational plan. The educational plan was a good lever, since the entire school team and parents were involved in preparing it.”* RM-47 *“The fact that the committee suggests new activities allows everyone to be involved in the projects. Theme weeks, class projects or a walk to the village centre—whatever the project, everyone gets on board and gets involved.”* RL-101 *“It was the support of the physical education teacher (leader), and her strong belief in the beneficial effects for students of playing sports and being active, that got everyone involved. She allowed teachers to personalize tools and provided ideas they could use. She called on educators at the daycare services as well as special education technicians at various times to implement activities for children. Through her actions, she influenced the school team.”* UM-270 ***Negative aspects*** *“Teachers have a lot to think about. As a leader, I didn’t assume a great enough leadership role during the program to encourage homeroom teachers and question their involvement.”* RL-7 *“There should have been more follow-up with homeroom teachers to increase their involvement.”* UL-19
**Resource mobilization** (including establishing new collaboration and/or **strengthening links with other organizations**) *3 subcategories:* -*Use of financial and material resources provided by the Ministry* -*Creation of new resources adapted to the context* -*Development of partnerships*	Total = 28% R = 26% U = 29% H = 30% M = 28% L = 27%	*“The funding received over the last three years allowed us to take the time to plan and carry out various activities. The purchase of additional equipment greatly enhanced what we already had.”* RM-60 *“It has been helpful for teachers to have access to the tools (videos, brain break stations) as well as the support of the resource person. Previously it was not a priority for them to take time to plan activities. Having someone do that part of the work/planning enabled them to implement everything without feeling like it was “extra work*.” UH-192 *“The level of engagement increased at the daycare services because they received more support (training and coaching) to explain the implementation of activities, which made their work easier.”* UH-176 *“The availability of resources such as FORCE 4 and specific programs through outside organizations (e.g., our partnership with the Canadian Ski Marathon for the school ski program) allowed us to improve the support offered in our pavilions.”* RL-99 *“Having a resource person available nearby to help teachers plan and organize more active games created a higher degree of engagement. The person also led discussions on the importance of physical activity for groups of students and staff members.”* UL-356
**Participation** *4 subcategories:* -*Participation of students in activities* -*Participation of students in setting up activities* -*Participation of staff in activities* -*Participation of staff in implementing the program*	Total = 25% R = 29% U = 23% H = 38% M = 22% L = 20%	*“I think that mobilization took place because we got together to discuss means and actions on a monthly basis. […] Many students also participate, which contributes to the staff’s engagement.”* RM-40 *“The increased level of engagement on the part of the homeroom teachers comes from their students’ engagement in active classes.”* RH-6 *“Members of the school team participate enthusiastically in the various activities proposed. In addition, homeroom teachers allow selected students to take time to help prepare activities and participate in them.”* UM-280 ***Negative aspects*** *“An increase in students arriving late for active mornings discouraged the staff, who attributed the lateness to students and parents not taking the activity seriously.”* UM-14 *“The homeroom teachers did not feel involved in the project, which sat squarely on the shoulders of the physical education teacher. They felt that the activities concerned them less. Since they were consulted very little over the first two years of the project, they withdrew somewhat from the proposed activities.”* UM-12 *“The first year, the involvement of teachers was mandatory, and they didn’t like the set-up.”* UM-13

*N*  number of participants,* SES*  socioeconomic status

^a^*R*  Rural,* U*  Urban,* H*  High SES, *M*  Middle SES,* L*  Low SES. Numbers refer to the school number

This first content analysis also revealed that the *local leadership* mechanism seemed relatively more triggered and/or more effective at generating outcomes in an urban context, whereas the *participation* and *assessment of the problem and critical awareness* mechanisms were more effective in the rural context (Table 4). Regarding SES differences, relatively fewer low-SES schools indicated the presence of mechanisms as a whole (Table 4), while a greater number reported negative outcomes, such as a decrease in the commitment of school team members to active time as implementation continued (9% of the low-SES participating schools, vs. 4% in middle-SES and 1% in high-SES).

### What worked for whom, and why?

The results of the quantitative analyses used to contextualize outcomes (*what worked for whom?*) and mechanisms (*why?*) as a function of the two main contextual variables (geographic and socioeconomic setting) are found in Table 5.

**Table 5:** What worked, for whom, and why? Outcome and mechanism variables in function of participating schools’ socioeconomic and geographic setting

	% of all schools	% of schools according to socioeconomic setting				% of schools according to geographic setting	
**OUTCOMES—all variables**	(*N* = 389)	**High SES**	**Middle SES**	**Low SES**	**Urban**		**Rural**
		(*N* = 89; U = 73; *R* = 16)	(*N* = 159; U = 100; *R* = 59)	(*N* = 141; U = 91, *R* = 50)	(*N* = 264)		(*N* = 125)
**Implementation of new PA routines**							
* Class: Active breaks*	94.5	94.2	96.2	92.6	94.4		94.6
* Class: Active learning* * activities*	76.3	76.7	79.0	75.6	75.4		78.3
* School: Active recesses*	85.9				84.3		89.1
*Urban*		84.7	81.0	87.4			
*Rural*		85.7^a, b^	82.5^b^	95.9^a^			
* School: Active assemblies*	80.6	80.2	83.4	76.5	81.7		78.3
* School: Active hallways*	74.1	76.7	72.0	75.0	74.6		72.9
* School: Outdoor field trips*	79.3	81.4	79.0	77.2	75.7		86.8*
* Daycare services:* * Implementation of* * new PA routines*	86.6				92.2		75.2***
*Urban*		97.2^a^	94.0^a, b^	87.4^b*^			
*Rural*		71.4	77.2	71.4			
**Staff engagement towards active time**							
* Homeroom teachers* * more engaged*	69.3	72.1	68.8	67.6	67.9		72.1
* Homeroom teachers* * less engaged*	4.5	1.2^a^	3.8^a, b^	7.4^b*^	4.1		4.7
* PE teachers more engaged*	61.5	70.9^a^	62.4^a, b^	55.1^b*^	60.8		62.8
* PE teachers less engaged*	0.0	0.0	0.0	0.0	0.0		0.0
* Daycare educators* * more engaged*	56.9	58.1	57.3	55.1	56.3		58.1
* Daycare educators* * less engaged*	0.3	0.0	0.0	0.7	0.7		0.0
**Perceived value of PA**							
* Greater perceived value* * of PA for its contribution* * to academic success*	84.9				84.3		86.0
*Urban*		91.7^a^	85.0^a, b^	79.3^b*^			
*Rural*		85.7^a, b^	80.7^b^	93.9^a*^			
**MECHANISMS—Selected variables**							
** Local leadership**							
* Leader maintained*	78.6				77.6		80.6
*Urban*		81.9	80.0	73.6			
*Rural*		64.3^a^	80.7^a, b^	87.8^b*^			
* Committee maintained*	60.5				64.2		52.7*
*Urban*		76.4^a^	67.0^a^	50.6^b**^			
* Rural*		42.9	57.9	49.0			
* Champions recognized*	48.1				48.1		48.1
*Urban*		56.9^a^	51.0^a, b^	37.9^b*^			
*Rural*		64.3	45.6	49			
** Resource mobilization and links with other organizations**							
* Strategies implemented* * to update activities*	56.2				55.6		57.4
*Urban*		55.6^a, b^	67.0^b^	46.0^a*^			
*Rural*		50.0	54.4	59.2			
* Partnerships formed with* * outside organizations*	33.5	31.4	34.4	35.3	30.2		40.3*
** Participation**							
* Students involved in* * setting up activities*	66.5				65.1		68.2
*Urban*		68.1	68.0	60.9			
*Rural*		50.0^a^	61.4^a^	79.6^b*^			

*SES*  socioeconomic status, *N*  number of participants, *U*  Urban, *R*  Rural. * indicates a significant difference (*p*-value < 0.05) between groups; ** indicates a significant difference (*p*-value < 0.01) between groups; *** indicates a significant difference (*p*-value < 0.001) between groups. In the case of differences according to socioeconomic settings, values on the same line with different superscripts (a, b) differ significantly at *p* < 0.05. ^a^To simplify the presentation, only mechanism variables showing significant differences have been included in the Table. Results are presented according to socioeconomic setting for rural and urban schools separately when there was an interaction between the two settings.

From an overall perspective, the vast majority of participating schools reported having implemented new PA routines in class, during recess, at the school and at the daycare services (between 74 and 95%, depending on the means) over the three years of program implementation. A majority (85%) of schools also saw their school team place greater value on PA for its contribution to academic achievement and observed an increase in commitment to active time on the part of homeroom teachers (69%), PE teachers (62%) and daycare educators (57%) over the three-year implementation period, despite the progressive decrease in funding.

Schools located in a rural setting integrated more outdoor field trips than did urban schools (87% vs. 76%, *p* < 0.05) but fewer new PA routines at daycare services (75% vs. 92%, *p* < 0.001). For all other outcomes, the quantitative analyses did not reveal any difference between urban and rural settings. Regarding variations in SES, the quantitative analyses support what was observed qualitatively: the commitment of school team members to daily active time is, on average, lower in underprivileged settings than at more privileged schools. In the former, fewer PE teachers became more engaged over the implementation period (55% vs. 71%, *p* < 0.05), while more homeroom teachers became less engaged (7% vs. 1%, *p* < 0.05). Despite the foregoing, rural schools in an underprivileged setting performed better than other rural schools by integrating more active recesses (96% vs. 86% and 83% for high and middle SES, p < 0.05) and by a greater increase in the value placed on PA by the school team for its contribution to academic success (94% vs. 86% and 81% for high and middle SES, *p* < 0.05). However, urban schools in an underprivileged setting performed negatively compared with other urban schools. They integrated fewer new PA routines at their daycare services (87% vs. 97% and 94% high and middle SES, *p* < 0.05), and a lower proportion of those schools reported an increase in the value placed on PA for its contribution to academic success (79% vs. 92% and 85%, for high and middle SES, *p* < 0.05).Regarding the mechanism variables, on the one hand, underprivileged rural schools outperformed middle- and high-SES rural schools for variables related to the *local leadership* and *participation* mechanisms, which may explain the relatively stronger outcomes in that setting (Table 5).

On the other hand, urban schools in an underprivileged setting performed more poorly than other urban schools for variables associated with the *local leadership* and *resource mobilization* mechanisms. A lower proportion of low-SES urban schools appointed and confirmed their intention to maintain a committee in charge of the program (51% vs. 76% and 67% for high and middle SES, *p* < 0.01), and a lower proportion of those schools identified and publicly acknowledged program champions in their school (38% vs. 57% and 51% for high and middle SES, *p* < 0.05). Compared with middle-SES schools, they were also fewer to have implemented strategies for updating the activities offered to students (46% vs. 67%, *p* < 0.05).

Finally, urban schools in general presented certain contextual factors that may limit the activation of mechanisms and affect the scope of the culture shift. On average, urban schools received less funding to implement the program ($53/student, SD = $25 vs. $73/student, SD = $39; *p* < 0.001; Table 3), while a larger proportion of urban schools reported experiencing resistance within the school team towards implementing daily active time (53% vs. 34%, *p* < 0.001, Table 3). Although not statistically significant, a higher proportion also reported a large turnover in homeroom teachers during implementation (22% vs. 14%, Table 3). The final qualitative analysis once again shows that in urban schools (context), local leadership (mechanism) appears to be relatively more important for generating outcomes than is the case in rural settings:

The fact that the principal made physical activity a priority in our educational plan was a very important factor in implementing change. Setting up a committee to act on that priority was also a factor. UH-68.

In underprivileged urban settings, several schools explained that a lack of resources (contextual factor) made it difficult to free up staff to lead the program and form a committee (*local leadership* mechanism) and, as a result, limited student and staff participation (*participation* mechanism), which appears to have compromised the sustainability of changes in practice (outcomes) in those settings (Table 6). A lack of time, heavy teacher workloads and managing students with special needs are other contextual factors identified by schools in an underprivileged setting that seem to have limited the activation of mechanisms (Table 6).

**Table 6:** Contextual factors (barriers and enablers) and CMO links in underprivileged rural and urban settings

**CONTEXT**	**Contextual factors**	% of schools	***Quotes—CMO links***
		(N _rural low SES_ = 48	
		N _urban low SES_ = 87)	
**Rural underprivileged**	**Barriers**		
	Staff turnover	Rural: 42% (vs Urban: 23%)	*“Staff mobility is one aspect that can have a negative impact. We have new teachers at the school every year. We’ve had good mobilization so far and hope that it continues.”* RL-74^a^
			*“Changing the person in charge of the program each year could compromise the sustainability of changes.”* RL-8
	**Enablers**		
	Presence of appropriate infrastructure and/or environments	Rural: 40% (vs Urban: 8%)	*“Improvements to the school yard that will be made this summer will make it possible to organize recesses for the students and offer a greater variety of outdoor activities.”* UL-27
			*“There are wooded areas nearby, the Parc régional des Appalaches, where we can go hiking.”* RL-73
	Presence of partnerships	Rural: 23% (vs Urban: 8%)	*“Collaboration with the community encourages the maintenance of projects involving physical activity (access to several municipal facilities, parks, swimming pool, dome, bike path, trails, *etc*.).”* RL-95
			*“The school’s partnership with the ski program during the winter. We are the first school to have tried this program and will continue this very fruitful partnership.”* RL-55
			*“The municipality’s financial support is very helpful.”* RL-11
	**Barriers**		
**Urban underprivileged**	Lack of financial resources and the end of funding	Urban: 44% (vs. Rural: 10%)	*“Reducing the funding that allows us to allocate time for physical education teachers to get students moving more threatens the sustainability of activities. These activities are the pillars of students’ active time, so if the physical education teachers are not available to help homeroom teachers by providing direction or turnkey activities, it’s clear that active time outside of physical education classes will almost disappear. […] Without the physical education teachers to oversee everything, few changes are introduced to encourage active time.” UL-123*
			*“Given that we are in an underprivileged area, as principal I work alone and there are specific issues related to the environment, the support of a project leader is required for the actions to be sustainable.”* UL-14
			*“In an underprivileged setting, the majority of our resources are used for basic needs, such as snacks, Breakfast Club, school supervision, special education technicians and remedial education. Resources provided under Measure 15,023 truly provide wonderful experiences outside the city where students can get moving and be active in the outdoors. […] The loss of funding will mean no more outdoor field trips.” UL-95*
			*“One major obstacle is the availability of enough quality winter clothing to hold outdoor activities, especially for grade 5 and 6 students, whose parents cannot always prioritize this type of expense for children who are often in a rapid growth phase.”* UL-78
	Lack of time, work overload and competition with other subjects, managing students with special needs	Urban: 9% (vs. Rural: 4%)	*“The belief that the program is demanding and that academic learning takes precedence over physical activity are, to some degree, obstacles to maintaining the changes.”* UL-39
			*“There are too many requests to get students moving. At some point, students need to be in class to learn.”* UL-17
			*“The need to manage poor behaviour during some activities led to a decrease in staff engagement.”* UL-20
	**Enablers**		
	The presence of a committee	Urban: 14% (vs. Rural: 6%)	*“Maintaining a working committee composed of teachers, PE teachers and the principal is what ensures implementation of the program at the school.”* UL-34
			*“The presence and involvement of the committee, without which the project could fail.”* UL-85

*N*  number of participants, *SES*  socioeconomic status, ^a^*R*  Rural; *U*  Urban; *H*  High SES; *M*  Middle SES; *L*  Low SES; Numbers refer to the school number

In rural settings (context), the participation mechanism appears to have been relatively more effective in generating outcomes. Changes in practice seem to have been initiated less by formal leadership (mechanism) and more by informal participation in various activities: “In our small school, all the staff got involved and participated actively” (RL-123). “The fact that teachers had informal discussions in the hallway motivated them to try new things” (RM-71). Access to infrastructure and quality outdoor environments, as well as the presence of partnerships, were also mentioned by underprivileged rural schools as contextual factors that enabled outcomes to be achieved. It is interesting to note that some contextual factors (such as partnerships and the presence of a committee) were previously identified as mechanisms (Table 4), which indicates that the community empowerment process appears to have modified, over the course of implementation, the context in which schools evolve.

## Discussion

The purpose of this study was to uncover what worked (and did not work), for whom, how, and why during the three-year implementation period of the *Active at School!* DPA program, while paying particular attention to the socioeconomic setting of the participating schools. The program’s flexibility appears to have enabled the activation of a community empowerment process within the school teams, leading to a cultural shift towards sustained DPA provision in high-SES and middle-SES schools as well as in low-SES rural schools. Contextual factors specific to schools in an underprivileged urban setting seem to have limited the activation of mechanisms, hindering a shift towards a sustained DPA provision in those schools, which raises concerns as to the medium- and long-term effects of the program on social inequalities with respect to PA and health. To better understand the differences between urban and rural underprivileged settings, it is necessary to consider the dynamic, adaptive and nonlinear nature of program implementation [36–38]. Our results suggest that the community empowerment process unfolded differently depending on the geographic and socioeconomic setting of schools, and that the different mechanisms do not appear to have been activated in the same way, in the same order or following a linear structure.

Firstly, our results suggest that, for all contexts, the *assessment of the problem* and *critical awareness* mechanisms played a pivotal role in program implementation and potential sustainability by creating a feedback loop [37] with the *participation* mechanism. The qualitative analysis shows that when school team members experienced positive changes in students, this reinforced the sense that the program was meeting a need, which encouraged staff to analyze their own practices, participate in identifying solutions, and even use the resources provided to further integrate active time. Participation, in turn, nourished a shared vision [18] of the importance of DPA routines in the school and fostered the commitment of staff to active time. The importance of observed benefits to implementation and sustainability of school-based PA programs has already been seen in previous research [18–20], so this result is not new. Our results do, however, further the existing literature by considering the dynamic nature of this mechanism and what enables its activation.

Our results suggest that, in urban settings, the *local leadership* mechanism is critical to initiating the community empowerment process and activating a feedback loop. It seems that urban schools require strong local leadership to begin the process, since those schools are, on average, larger than rural schools, present greater resistance to program implementation and have a greater turnover of homeroom teachers (Table 3). In urban settings, therefore, formal leadership structures (leader, committee and follow-up meetings) appear to have enabled the mobilization of resources, as well as support and monitoring for members of the school team in order to ensure their participation. That participation appears to have then allowed staff to experience the benefits of active time, which reinforced their participation (feedback loop).

In underprivileged urban settings, however, our results show that the *local leadership* mechanism was less activated than in high- and middle-SES urban schools, which seems, in turn, to have limited activation of the feedback loop. Indeed, a smaller proportion of low-SES urban schools set up a committee and expressed their intention to maintain it, and a smaller proportion identified and publicly recognized champions in their school (Table 5). In addition, the qualitative analyses show that in the absence of ongoing external funding for the program, underprivileged urban schools are not able to sustain local leadership structures associated with the integration of active time, which makes them vulnerable to funding being cut off (Table 6).

The lack of resources in this setting and teachers’ heavy workload are contextual barriers identified by underprivileged urban schools that may explain, in part, the challenges schools face in implementing and maintaining new local leadership structures. Some schools explained that their disadvantaged context limits the reallocation of school resources towards priorities other than children’s basic needs (Table 6).

This need for additional financial support for schools in disadvantaged areas has already been underscored in the literature. Peralta et al. [39] showed that low-SES schools consistently report more barriers and fewer enablers to PA than their high-SES counterparts, while their needs are already greater. Indeed, lack of PA is usually more prevalent among lower socioeconomic groups and often passes from generation to generation [40]. To tackle social health inequities, it has therefore been suggested that public action should consider higher investment for disadvantaged schools [40]. This approach to promoting equal opportunities is called proportionate universalism [9] and consists in “offering universal interventions intended for all [environments], but with modalities or intensity that vary according to needs” ([16], p. 14, *unofficial translation*).

Consideration of the geographical context in our study therefore made it possible to show that in rural settings, an underprivileged context does not seem to have negatively affected implementation of the program. There, unlike the situation with urban schools in an underprivileged setting, the program seems to have initially activated the participation mechanism, triggering a feedback loop from the outset, as can be seen from the benefits (Fig. 1). This phenomenon seems to have facilitated the activation of other mechanisms, enabling the entire community empowerment process to become self-sustaining and leading to a cultural shift in favour of sustained DPA provision. This result is surprising given that a recent systematic review [41] highlighted that school-based PA interventions conducted in rural settings may pose greater challenges than in urban settings. A possible explanation for our result might be community involvement, a component that was missing from all rural intervention included in the review [41] but appeared to be a significant mechanism for the underprivileged rural schools included in our study. Indeed, our results highlighted that in underprivileged rural settings, the mobilization of nearby resources (such as strengthening links between schools and other organizations in the community and reinforcing the use of nearby infrastructure and environments) appears to have successfully modified the context (Fig. 1) in which schools evolve and created the foundations required to maintain changes in practice over the long term. This modification of the context through the mobilization of nearby resources was not observed in underprivileged urban settings (Fig. 2). Since developing partnerships have been identified several times in the literature as a factor favouring the implementation and sustainability of school-based PA programs [18, 42], additional support for schools in underprivileged urban settings aimed at enhancing community partnerships would be an avenue to explore for supporting sustainable implementations of the program. Rural underprivileged schools also demonstrated a greater involvement of students in setting up activities (Table 5). Student involvement in setting up activities has been identified in the literature as a factor favouring the implementation and sustainability of school-based PA programs [18, 19]. Hence, this could be another avenue to explore in urban underprivileged settings to drive the process of change. Nonetheless, due to the nature of their context, it remains that allocating additional financial resources in urban underprivileged settings appears necessary to enable schools to adopt and sustain the leadership structures that allow them to build or solidify partnerships in their community and/or involve more students.

### Limitations

These findings enrich the understanding of the underlying mechanisms at play during the implementation of school-based DPA, but it is important to acknowledge limitations. First, the participating schools were self-selected to enroll in the program and were not representative of all schools in the province, limiting the generalizability of the findings. Second, the surveys provided self-reported implementation data and may be subject to bias on behalf of schools. Finally, we were not able to interview participants, which may have deprived us of some nuances that in-person interviews could have offered. Nonetheless, using questionnaires to collect large amounts of qualitative and quantitative data allowed for both an overall and a refined analysis of the implementation processes.

## Conclusion

The purpose of this study was to evaluate the implementation of the DPA program *Active at School!* using a realist approach, while paying particular attention to the socioeconomic setting of the participating schools. Our realist evaluation showed that a DPA program that gives local communities (the school teams, in this case) autonomy in the choice of strategies appropriate to their situation, while providing financial and material support, fosters the emergence of an organizational culture shift towards supporting a sustained DPA provision. The community empowerment domains constitute an insightful framework for identifying settings with greater needs and pointing to mechanisms that drive local change processes. It appears that contextual factors specific to schools located in underprivileged urban settings make the implementation of the DPA program more difficult, with the risk of accentuating existing social inequalities in health if additional resources are not deployed. Policy-makers should consider adjusting resource allocations to meet the needs of schools when implementing health-related policies in order to encourage the establishment of environments that can support all children in adopting a healthy lifestyle. [34] 

## Supplementary Information

**Additional file 1.** List of items to be included when reporting realist evaluations (Wong et al., 2016)

## Data Availability

The datasets generated and/or analysed during the current study are available from the corresponding author on reasonable request.

## Abbreviations

PA: Physical activity
DPA: Daily physical activity
PE: Physical education
SES: Socioeconomic status
CMO: Context-mechanism-outcome
