# Correction to: Do the implementation processes of a school-based daily physical activity (DPA) program vary according to the socioeconomic context of the schools? A realist evaluation of the *active at school* program

**DOI:** 10.1186/s12889-022-12968-6

**Published:** 2022-03-16

**Authors:** Véronique Gosselin, Suzanne Laberge

**Affiliations:** School of Kinesiology and Physical Activity Sciences, Université de Montréal, C.P. 6128, Succursale Centre-Ville Montréal, Québec, H3C 3J7 Canada

**Correction to: BMC Public Health 22, 424 (2022).**

**https://doi.org/10.1186/s12889-022-12797-7**

During the publication process of the original article an error was introduced in the results section. This error is shown below for clarification. The original article has been updated.

1. The bold sentence was missing:
  - **The final qualitative analysis once again shows that in urban schools (context),** ***local leadership*** **(mechanism) appears to be relatively more important for generating outcomes than is the case in rural settings:**

The fact that the principal made physical activity a priority in our educational plan was a very important factor in implementing change. Setting up a committee to act on that priority was also a factor. UH-68.

